# A Rapid and Accurate Method to Evaluate *Helicobacter pylori* Infection, Clarithromycin Resistance, and CYP2C19 Genotypes Simultaneously From Gastric Juice

**DOI:** 10.1097/MD.0000000000003458

**Published:** 2016-05-27

**Authors:** Chao-Hung Kuo, Chung-Jung Liu, Ching-Chia Yang, Fu-Chen Kuo, Huang-Ming Hu, Hsiang-Yao Shih, Meng-Chieh Wu, Yen-Hsu Chen, Hui-Min David Wang, Jian-Lin Ren, Deng-Chyang Wu, Lin-Li Chang

**Affiliations:** From the Division of Gastroenterology, Department of Internal Medicine (C-HK, C-JL, H-MH, H-YS, M-CW, D-CW), Kaohsiung Medical University Hospital, Kaohsiung, Taiwan; Department of Medicine, Faculty of Medicine, College of Medicine (C-HK, H-MH, Y-HC, D-CW), Kaohsiung Medical University, Kaohsiung, Taiwan; Center for Stem Cell Research (C-HK, H-MW, D-CW), Kaohsiung Medical University, Kaohsiung, Taiwan; Department of Microbiology and Immunology (C-CY, L-LC), Kaohsiung Medical University, Kaohsiung, Taiwan; School of Medicine, College of Medicine (F-CK), E-Da Hospital, I-Shou University, Kaohsiung, Taiwan; Department of Internal Medicine (H-YS, M-CW, D-CW), Kaohsiung Municipal Ta-Tung Hospital, Kaohsiung, Taiwan; Division of Infectious Diseases, Department of Internal Medicine (Y-HC), Kaohsiung Medical University Hospital, Kaohsiung, Taiwan; Department of Fragrance and Cosmetic Science (H-MW), Kaohsiung Medical University, Kaohsiung, Taiwan; and Department of Gastroenterology (J-LR), Zhongshan Hospital affiliated to Xiamen University, Xiamen, Fujian, China; Center for Infectious Disease and Cancer Research (D-CW), Kaohsiung Medical University, Kaohsiung, Taiwan.

## Abstract

Because *Helicobacter pylori* (*H pylori)* would cause carcinogenesis of the stomach, we need sufficient information for deciding on an appropriate strategy of eradication. Many factors affect the efficacy of eradication including antimicrobial resistance (especially clarithromycin resistance) and *CYP2C19* polymorphism. This study was to survey the efficiency of gastric juice for detecting *H pylori* infection, clarithromycin resistance, and CYP2C19 polymorphism.

The specimens of gastric juice were collected from all patients while receiving gastroscopy. DNA was extracted from gastric juice and then *urease A* and *cag A* were amplified by polymerase chain reaction (PCR) for detecting the existence of *H pylori.* By PCR-restriction fragment length polymorphism (PCR-RFLP), the *23S rRNA* of *H pylori* and *CYP2C19* genotypes of host were examined respectively. During endoscopy examination, biopsy-based specimens were also collected for rapid urease test, culture, and histology. The blood samples were also collected for analysis of *CYP2C19* genotypes. We compared the results of gastric juice tests with the results of traditional clinical tests.

When compared with the results from traditional clinical tests, our results from gastric juice showed that the sensitivity (SEN), specificity (SPE), positive predictive value (PPV), negative predictive value (NPV), and accuracy to detect *H pylori* infection were 92.1% (105/114), 92.9% (143/154), 90.5% (105/116), 94.1% (143/152), and 92.5% (248/268), respectively. The SEN, SPE, PPV, and NPV to detect clarithromycin resistance were 97.3% (36/37), 91.5% (43/47), 90.0% (36/40), and 97.7% (43/44), respectively. By using PCR-RFLP, the consistency of human *CYP2C19* gene polymorphism from blood samples and gastric juice was as high as 94.9% (149/157).

The manipulated gastric juice is actually an effective diagnostic sample for evaluation of *H pylori* existence, clarithromycin resistance, and host *CYP2C19* polymorphism.

## INTRODUCTION

It is well known that *Helicobacter pylori (H pylori)* is the cardinal pathogen of many gastrointestinal disease including gastritis, peptic ulcer, and gastric cancer.^1^ In Taiwan, the average prevalence rate of *H pylori* is around 54% and it is higher in elderly population.^2,3^ So it is important to accurately diagnose and successfully eradicate the *H pylori*.

In recent years, the successful eradication rate of first-line triple therapies has decreased to <80%.^4,5^ There are many factors resulted in the failure of eradication including antibiotic resistance, the cytochrome P450 2C19 (*CYP2C19*) polymorphism, and poor compliance, etc.^6^ According to previous studies, the main cause of eradicating failure was antibiotic resistance. To overcome the antibiotics resistance, we must use the suitable agents according to the local results of resistance.^7^ Clarithromycin resistance is the most important issue among these challenges.^8^ Traditional triple therapy is not suitable as the choice of an empirical first-line therapy because the increase in the prevalence of clarithromycin- and metronidazole-resistance.^9,10^ Therefore, the Maastricht IV Consensus has recommended that clarithromycin should not be used in areas with >15% *H pylori* clarithromycin-resistant strains.^9^

Traditionally, bacteria must be cultured first, and then susceptibility to antimicrobial agents is determined by tests (for example: E test). However, there are many challenges for this procedure. First, it takes nearly 1 week for the physician to determine whether appropriate antimicrobial agents were used to treat the disease. Second, the success rate of culturing is not high (60%–70%). Third, this procedure needs a laboratory team; accordingly, the physicians need new methods to overcome these problems.

The principal enzyme implicated in the metabolism of proton pump inhibitors (PPIs) (e.g., omeprazole) is *CYP2C19*.^11,12^ The polymorphisms of *CYP2C19* would also influence the outcome of *H pylori* eradication. Several trials have been published concerning the effect of the *CYP2C19* genotype on eradication of *H pylori* by various PPI-based therapies.^5,13–15^ There is obvious difference composition of *CYP2C19* gene polymorphism between Asian people and European Caucasians. Higher ratio of poor metabolizers is noted in Asian people. So it is more important to survey the *CYP2C19* genotype especially for patients receiving rescue therapies.

Gastric juice has been reported as efficacious for the detection of clarithromycin-resistant *H pylori*.^16,17^ The aim of this study was to survey the efficiency of gastric juice for detecting clarithromycin resistance and *CYP2C19* polymorphism, and compare the accuracy with traditional methods.

## METHODS

### Patients

Enrolled patients were those who visited the gastroenterological clinic of Kaohsiung Medical University Hospital (KMUH) between June 2013 and March 2015 with the complaint of dyspepsia. Our study was matched all the criteria of the Declaration of Helsinki^18^ and was approved by the KMUH ethics committee. Exclusion criteria included ingestion of antibiotics, bismuth, or PPI within the prior 4 weeks; patients with allergic history to the medications used; patients with previous gastric surgery; the coexistence of serious concomitant illness (e.g., decompensated liver cirrhosis, uremia); and pregnant women.

### Study Design

During the examination of gastroendoscopy, we inserted the suction tube via the working channel of endoscope. Then we aspirated the gastric juice (3–4 mL) from the fundus area of stomach. DNA was extracted from gastric juice and then detection of *urease A* and *cag A* was performed by PCR representing the existence of *H pylori.* At the same time, PCR-RFLP was used to detect the point mutations in 2 positions (A to G at 2142, 2143) in the 23S rRNA gene that is associated with clarithromycin resistance in *H pylori*. The biopsy specimens were obtained from patients undergoing gastroscopy, and were then cultured. The *H pylori* strains were tested for antibiotic susceptibility using the E test. The results of gastric juice samples were compared with the results of the E test.

**FIGURE 1:**
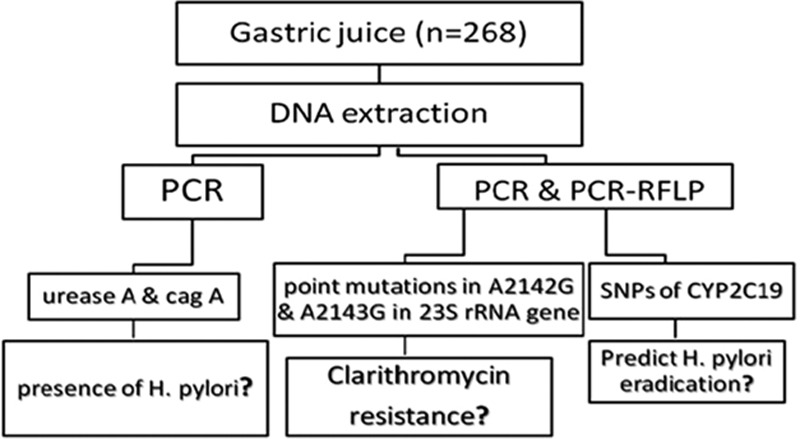
Flowchart of study design.

In addition, *CYP2C19* m1 and m2, which are associated with *CYP2C19* genetic polymorphisms in humans, were determined by PCR-RFLP. The results of gastric juice samples were compared with the analysis of *CYP2C19* genetic polymorphisms via blood samples (Figure 1).

### Confirmation of *H pylori* Infection

Patients might also receive the ^13^C-urea breath test (UBT) to detect *H pylori*. The infection status of *H pylori* infection was considered positive if the results met the following criteria (clinical gold standard): positive culture, positive UBT, and concordant positive results in both histology and rapid urease test (RUT). If the patients presented only RUT positive or histology positive, we regarded them as indistinct cases.

### Extraction of Genomic DNA From Gastric Juices, Blood Sample, and *H pylori* Stains

Gastric juice was obtained during the gastroscopy. Before storage, the pH of gastric juice (pH value ≤ 3) was adjusted to >3 with culture medium DMEM (Invitrogen) for improving DNA quality in gastric juice and prevent enzyme inhibition in further PCR reaction. The ratio of gastric juice to DMEM was 1:2. The gastric juice/culture medium mixture was then centrifuged at 12,000 rpm for 10 minutes to precipitate the gastric mucus for purifying genomic DNA. DNA from the enrolled patients’ blood samples, gastric juices that included host cells with/without *H pylori*, and *H pylori* stains were extracted with the DNeasy Blood & Tissue kit (QIAGEN, Germany), and then was purified by Wizard SV and PCR Clean-up systems (Promega, Madison, WI). DNA products were then subjected to PCR and PCR-RFLP. PCR of *urease A* and *cag A* in gastric juices were used to identify the *H pylori* infection. PCR-RFLP of *H pylori* 23S rRNA point mutations (A2142G and A2143G) was used to measure the clarithromycin resistance of *H pylori*. PCR-RFLP of *CYP219 m1, m2* was used to measure the *CYP2C19* polymorphism of host.

### Polymerase Chain Reaction

PCR of *urease A* and *cag A* genes of *H pylori* from gastric juice was used to measure the reality of *H pylori* infection status. PCR was well performed with the purified genomic DNA as the template. The sequence information of primers including *ureas*e *A* and *cag A* virulence factors was listed in the Table 1. DNA extracted product from gastric juice (40 ng) was diluted with the PCR buffer (50 mM KCl, 10 mM Tris–HCl, and 2 mM MgCl2) to a final volume of 20 mL, containing 0.5U of Taq DNA polymerase (Invitrogen), 0.25 μM dNTPs master mix (Yeastern Biotech, Taiwan) and 0.5 μM primer. For each experiment, up to 60 cycles were performed in a Bio-Rad thermal cycler (Bio-Rad). PCR was initiated with a hot start (1 minute at 95°C), the samples were then subjected to 60 cycles at 95°C for 1 minute, 48°C for 1 minute, and 72°C for 1 minute. The PCR products (*urease A*: 414 bp, and *cag A*: 349 bp) were analyzed on 2% agarose gel electrophoresis and the Kodak Scientific 1D Imaging System (Eastman Kodak Company, New Haven, CT).

**TABLE 1:**
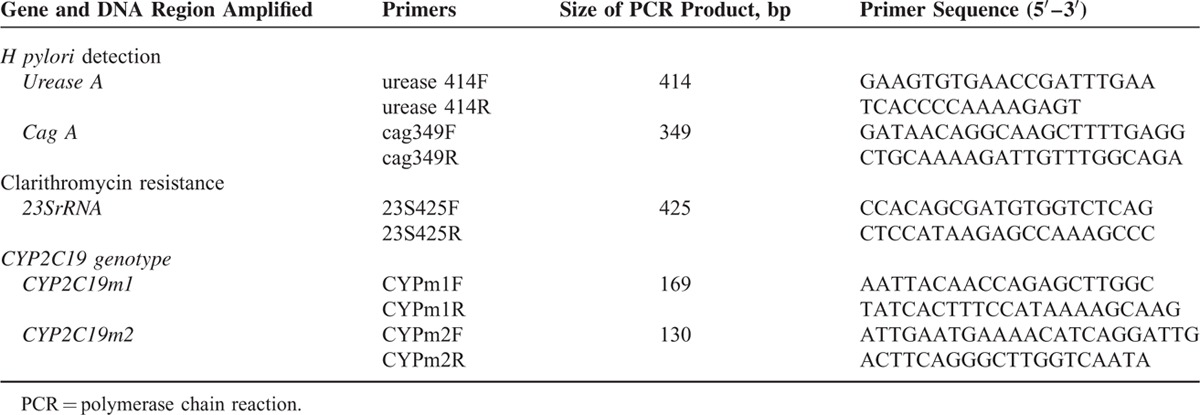
Primers Used in Detecting *H pylori*, Clarithromycin Resistance, and CYP2C19 Genotypes

### Polymerase Chain Reaction-restriction Fragment Length Polymorphism

PCR-RFLP of *H pylori* 23S rRNA point mutations (A2142G and A2143G) was used to measure the clarithromycin resistance of *H pylori*. PCR-RFLP of *CYP219 m1, m2* was used to measure the *CYP2C19* polymorphism of host. The primer sequences are listed in Table 1. For *H pylori* 23S rRNA point mutations (A2142G and A2143G) analysis, PCR products were digested in 10 μL of the reaction buffer containing *MboII* and *Bsa I* restriction enzymes (BioLabs, New England). The digested fragments were separated by 3% agarose gel electrophoresis. A2142G point mutation of *H pylori* 23S rRNA showed the 332- and 93-bp products. A2143G point mutation of *H pylori* 23S rRNA showed the 319- and 106-bp products.

**FIGURE 2:**
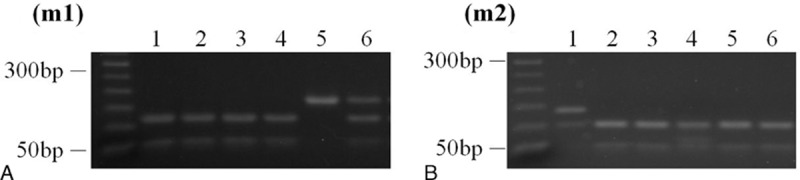
PCR products from gastric juices of 6 patients were digested with (A) Sma I (m1, 120 + 49 bp), or (B) Bam HI (m2, 96 + 34 bp). Lane 1: *CYP2C19* genotype was wt/m2 (het-EM). Lanes 2–4: *CYP2C19* genotype was wt/wt (hom-EM). Lane 5 *CYP2C19* genotype was m1/m1 (PM). Lane 6: *CYP2C19* genotype was wt/m1 (het-EM). PCR = polymerase chain reaction.

For *CYP2C19* genotype analysis, PCR products were digested with *SmaI* and *BamHI* restriction enzymes (BioLabs, New England) in 10 μL of the reaction buffer at 25°C overnight. The digested fragments were separated by 3% agarose gel electrophoresis. The types were assigned by the appearance of 2 digestion bands: 120, 49 bp for *CYP2C19* m1; and 96, 34 bp for *CYP2C19* m2 (Figure 2).^19,20^

### *H pylori* Culture and Antimicrobial Resistance

Gastric biopsy specimens were collected and rubbed on the surface of CDC Anaerobe 5% Sheep Blood Agar (Campy-BAP agar plate; Brucella agar + 10% whole sheep blood + IsoVitalex) (BD Diagnostics). Then, plates were incubated at 37°C with microaerobic condition (10% CO_2_, 5% O_2_, and 85% N_2_) for 5 days for *H pylori* colony isolation. Isolates of *H pylori* were further confirmed on the bases including the positive urease, catalase, and oxidase biochemical reactions; and the 16S rRNA presence. Following successful culturing, the *H pylori* strains were tested for antibiotic susceptibility using the E test. Standard reference *H pylori* strain ATCC 43504 was used as control in the present study. *H pylori* strains with a minimal inhibitory concentration (MIC) value >1 mg/L were considered resistant to clarithromycin.^21^

### Sequencing

PCR products of genomic DNA from gastric juices were sequenced for *urease A* gene for confirmation the reality of *H pylori* infection. Standard reference ATCC *H pylori* 43504 strain was used as control. DNA sequencing was carried out using an Applied Biosystems 3730XL DNA sequencer, and sequences were analyzed by Chromas version 2.23 (Genomics BioSci & Tech. Co., Ltd, Taiwan).

### ^13^C-UBT

We used the test material (^13^C-urea) (the Institute of Nuclear Energy Research, Taiwan) for every patient. Our test meal was fresh whole milk (100 mL). The detailed process was according to manual from the Institute of Nuclear Energy Research and reported in our previous study.^22^

### Histological Examination

Gastric biopsy specimens were fixed with 10% formalin and were then embedded in the paraffin. Sections (3∼4 μm) were obtained by using a Lica 2145 rotary microtome (Leica Microsystems, Nussloch, Germany) and were then followed by the Giemsa staining procedure for the detection of *H pylori*.

### Rapid Urease Test

The Campylobacterlikeorganism (CLO) test is a well-known RUT for the diagnosis of *H pylori*. The results of the CLO test (Delta West Bentley, WA, Australia) were interpreted as positive if the color of the gel turned pinkish red 6 hours after examination at room temperature.

### Statistical Analysis

We analyzed the collected data using the statistical software package STATA. In this study, χ^2^ test was chosen for statistical analysis. *P* < 0.05 was considered to be statistically significant.

## RESULTS

In patients with *H pylori* infection, we could extract DNA of *H pylori* from gastric juice for detecting gene expression of *urease A* and *cag A*. Usually, *urease A* could be identified by a band on 414 bp. *cag A* could be identified by a band on 349 bp.

We show the impact of pH value on the performance of PCR; the PCR cannot be performed in the environment of pH ≤ 3. Our result disclosed that PCR for detecting *H pylori* infection in pH >4 could only be carried out after neutralization by DMEM Eagle's medium. Furthermore, this step could maintain the high efficiency of DNA extraction from gastric juice that had even been stored at 4°C for 7 days. Therefore, it is convenient for physicians transferring the samples to the laboratory center.

There were 268 patients enrolled in this study. One hundred and fourteen patients showed positive results of *H pylori* infection according to traditional clinical gold standards. After comparing the gastric juice-PCR amplification (*urease A/cag A*) with the clinical gold standard, our results showed that the gastric juice test had 92.1% (95% CI: 88.9–95.3) sensitivity (SEN) (105/114), 92.9% (95% CI: 89.8–96) specificity (SPE) (143/154), 90.5% (95% CI: 87–94) positive predictive value (PPV) (105/116), 94.1% (95% CI: 91.3–96.9) negative predictive value (NPV) (143/152), and 92.5% (95% CI: 89.3–95.7) accuracy (ACC) (248/268) in detecting *H pylori* infection (Table 2).

**TABLE 2:**
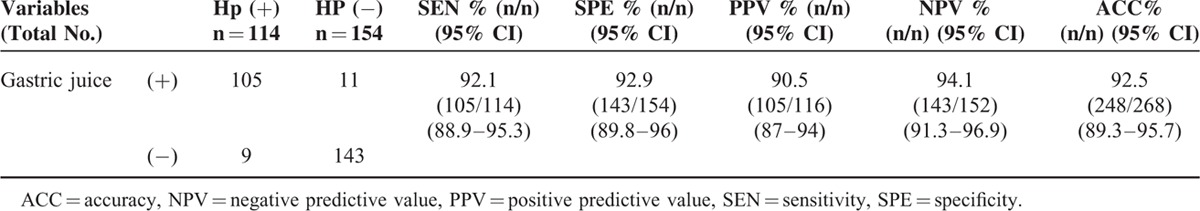
Compare Detection From Gastric Juice With Clinical Gold Standard

Most cases showed consistent results in both clinical gold standards and gastric juice methods. There were 9 patients presenting as positive according to the clinical gold standard but negative in the gastric-juice test (Table 2). We found that these patients’ UBT values all were around 4 to 6 (borderline value). Thus, we tried to follow the results of UBT 2 months later: 5 patients were validated as being without *H pylori* infection. These data demonstrated that the gastric juice test has less false-positive results.

Eleven patients were regarded as “indistinct case” when traditional clinical gold standard were used; however, the gastric juice test in these indistinct cases showed positive results of *H pylori* infection (Table 3). We further confirmed the status of *H pylori* infection in 8 of these 11 patients (8/11) by performing *urease A* gene sequencing via gastric juice. We compared the results of *urease A* gene sequence with *H pylori* ATCC 43504 standard strain. The *urease A* gene sequence of these 8 samples revealed high consistency (>96 %) with standard strain *H pylori* ATCC 43504. So we regarded these 8 patients as being infected by *H pylori*. In addition, 7 patients among these 11 patients received UBT. The results showed 3 patients were positive and 4 patients were negative in the UBT test 2 years later (Table 3).

**TABLE 3:**
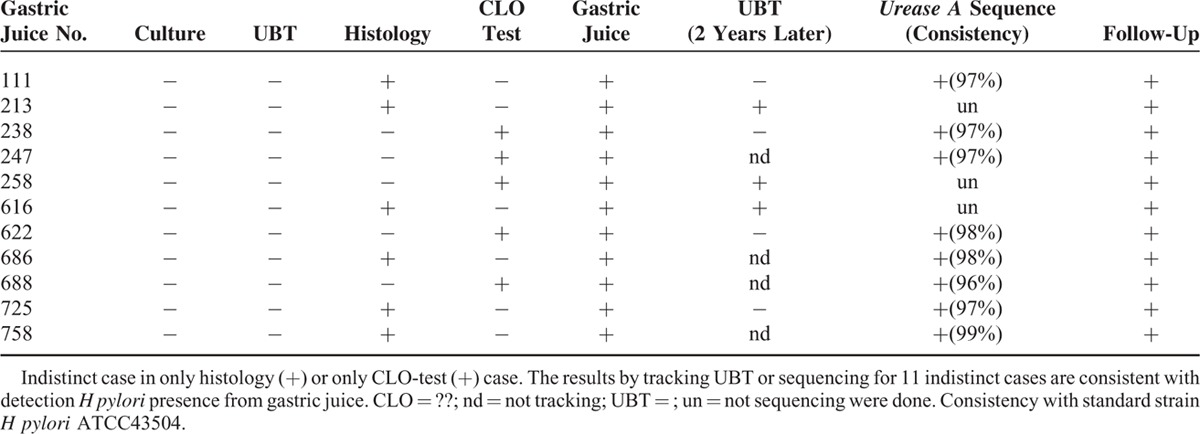
Follow-Up of the Infection of *H pylori* among Indistinct Cases (n = 11)

Among 114 *H pylori*-infected patients according to the clinical gold standard, *H pylori* was successfully cultured from 84 patients. The clarithromycin resistance in 37 isolates was found by E test, which were used as the control. We further analyzed the clarithromycin resistance from gastric juice by the PCR-RFLP method in these 84 patients. After comparison with the E test, the results of PCR-RFLP showed sensitivity of 97.3% (36/37), specificity of 91.5% (43/47), positive predictive value (PPV) of 90.0% (36/40), negative predictive value (NPV) of 97.7% (43/44), and accuracy of 94.0% (79/84) (Table 4). Most of the clarithromycin resistance cases showed the mutation of A2143G of 23S rRNA; one case showed the mutation of A2142G of 23S rRNA.

**TABLE 4:**
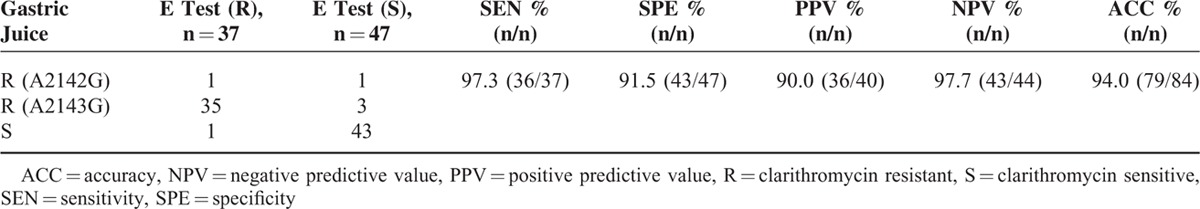
Comparison PCR-RFLP and E test to Detect Clarithromycin Resistance

Among 114 patients with *H pylori*-infection according to the clinical gold standard, 30 patients showed negative results in *H pylori* agar culture. Nine patients with borderline UBT results were excluded, while the other 21 patients among these 30 patients received further analysis. Importantly, our results showed that all of these 21 patients could be successfully surveyed for the susceptibility of clarithromycin from gastric juice. Nine among these 21 patients showed *H pylori* clarithromycin resistance due to A2143G mutation in 23S rRNA, while the other 12 patients showed clarithromycin sensitivity. It indicated that PCR-RFLP from gastric juice can replace the traditional E test to detect clarithromycin susceptibility in *H pylori* noncultured samples.

We analyzed the *CYP2C19* polymorphism of 157 patients, and compared the consistency between specimens from gastric juice test or blood. When blood specimens for *CYP2C19* polymorphism were used as standard, all samples from gastric juice (157/157) could successfully detect the *CYP2C19* genotypes. In addition, the consistency between these 2 tests was 94.9 % (149/157) (Table 5).

**TABLE 5:**
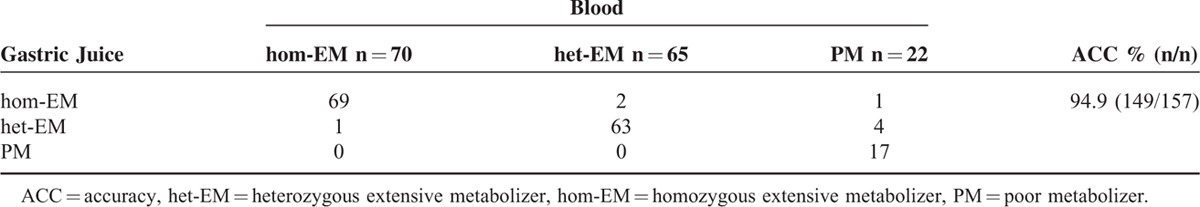
The Accuracy of PCR-RFLP From Gastric Juice in Analysis of CYPC219 Genotypes

## DISCUSSION

Our study developed the reliable method “gastric juice test,” which could rapidly and correctly survey *H pylori* infection, resistance of clarithromycin, and genotypes of *CYP2C19*. Our results revealed that gastric juice test provided rapid diagnosis, high sensitivity, specificity, and accuracy. This method could be easily carried out within 2 days. Besides these, the gastric juice sample could be preserved for 1 week after adding DMEM Eagle's medium, so we could easily manage the sample by PCR or PCR-RFLP within 1 week. It made this method convenient and reliable for distant cooperation. Because gastric juice can be collected in a simple manner without the risk of bleeding, it is preferred, relative to cultured isolates.^16^

There are many known virulent factors of *H pylori.* All *H pylori* strains in the world have *urease A*. In Taiwan, >95% of *H pylori*-infected patients show *cag A* positive,^23^ so we chose *urease A* and *cag A* as indicators of *H pylori* infection in this study. Previous studies have used *urease A* as indicator by nested PCR assay to survey the infection of *H pylori*.^24,25^ They also analyzed the A2142G and A2143G point mutation at *23S rRNA* by the same method. The nested PCR method was based on PCR and used 2 primer pairs. This method has higher specificity than PCR but also has higher possibility of cross-contamination. The nested PCR also needs a more complex procedure, has higher cost, and is more time-consuming, so most researchers prefer PCR for these analyses. One study has used gastric juice for analysis of *H pylori* infection;^26^ however, they did not extend the method to survey resistance of clarithromycin and genotypes of *CYP2C19*.

In clinical practice, physicians often meet the situation where patients show only 1 positive result of histology or RUT. This brings about difficulty in deciding on the best therapeutic strategy. Our results showed that these patients might actually be *H pylori*-infected. The possible causes of false-negative results in clinical tests might result from sampling error or lower bacterial load. The distribution of *H pylori* in the stomach would change after unsuccessful eradication.^27^ In addition, it is very important to know the strain resistance status for rescue therapies, as these disadvantages might be avoided in the gastric juice test, as gastric juice covers almost all the stomach area. Therefore, it is reasonable that the gastric juice test is more suitable for patients needing rescue therapy.

Current available *H pylori* tests have many disadvantages. The 13C-UBT has high sensitivity and accuracy, but is expensive. On the other hand, physicians would raise doubts when the results of RUT and histology are not consistent. In the past, this situation might be regarded as negative for *H pylori* infection; therefore, these false-negative results would mislead physicians to make the wrong decision.

The culture method is very specific for identification of *H pylori* infection. However, there are several disadvantages of the traditional E test. First, the MIC and the inhibitory zone of clarithromycin in the E test are decided by subjective observation, so this might result in errors in cases of borderline data. Second, the culture rate of *H pylori* is often not high. The gastric juice test has objective results and can almost perfectly analyze the susceptibility of clarithromycin. Compared with the culture method, the sensitivity and specificity of the gastric juice test were 100.0% and 95.7%, respectively.^28^ It means that the gastric juice test is reliable for susceptibility test of *H pylori*. This is a very important finding for second-line or rescue therapies. Besides this, the gastric juice test provided a faster report than did the culture method. Usually, we could know the result of the gastric juice-based test within 2 days.

It is also important to know the *CYP2C19* genotypes of the host, especially for those patients who would receive rescue therapies of *H pylori* infection. According to our results, the gastric juice test was also a useful and reliable method for analysis of *CYP2C19* genotypes. It showed high consistency with the blood sample test, so patients could know their status of *H pylori* infection, clarithromycin resistance, and *CYP2C19* genotypes at the same time when gastric juice was obtained under endoscopy examination. This change is beneficial for both patients and doctors.

Besides the accuracy and efficacy, the gastric juice test is also cheaper than traditional methods in Taiwan when we want to confirm the infection and susceptibility of *H pylori* (around 12 USD vs 120 USD). We also can get the results in 4 days by the gastric juice test. The duration is obviously shorter than traditional tests (around 10–12 days).

Our study had limitations. One limitation was that there might be mixed bacterial flora in the stomach. However, we did not check the efficiency of examining flora other than *H pylori*. Another possible limitation was that the clarithromycin-resistant and -susceptible *H pylori* might co-exist in some patients. However, we did not observe this phenomenon. The clarithromycin-resistant *H pylori* might be overlooked if clarithromycin-susceptible *H pylori* is dominant.^28^

## CONCLUSIONS

The manipulated gastric juice might actually be a more effective and economic diagnostic sample for evaluation of *H pylori* existence, clarithromycin resistance, and host *CYP2C19* gene polymorphism. It provides physicians with another tool to give the correct medications to treat *H pylori* infection as soon as possible, and would improve the eradication rate of *H pylori* infection. Accordingly, we suggest that it would be advantageous if this test were to be widely carried out, especially for those cases needing rescue therapies.
